# Gender, Marital Status, and Children as Risk Factors for Burnout in Nurses: A Meta-Analytic Study

**DOI:** 10.3390/ijerph15102102

**Published:** 2018-09-25

**Authors:** Guillermo A. Cañadas-De la Fuente, Elena Ortega, Lucia Ramirez-Baena, Emilia I. De la Fuente-Solana, Cristina Vargas, Jose Luis Gómez-Urquiza

**Affiliations:** 1 Faculty of Health Sciences, University of Granada, Avenida de la Ilustración N. 60, 18016 Granada, Spain; gacf@ugr.es (G.A.C.-D.l.F.); jlgurquiza@ugr.es (J.L.G.-U.); 2 Faculty of Psychology, University of Almería, Carretera Sacramento S.N., 04120 La Cañada, Almería, Spain; elenaortega@ual.es; 3 Brain, Mind and Behaviour Research Center (CIMCYC), University of Granada, Campus Universitario de Cartuja S.N., 18011 Granada, Spain; edfuente@ugr.es; 4 Faculty of Psychology, University of Valencia, Avenida de Blasco Ibáñez N. 13, 46010 Valencia, Spain; varpe@uv.es

**Keywords:** burnout, Maslach Burnout Inventory, meta-analysis, nurses, sociodemographic risk factors

## Abstract

The correlation between the burnout syndrome and sociodemographic variables in nursing professionals has been widely studied though research results are contradictory. The aim of this study was to assess the impact of gender, marital status, and children on the dimensions of the burnout syndrome (emotional exhaustion, depersonalization, and personal accomplishment) in nursing professionals, as measured with the Maslach Burnout Inventory. The search was performed in May 2018 in the next databases: CINAHL, CUIDEN, Dialnet, Psicodoc, ProQuest Platform, OVID Platform, and Scopus with the search equation (“Maslach Burnout Inventory” OR “MBI”) AND “nurs*”, without using any search restriction. The sample was *n* = 78 studies: 57 studies for gender; 32 for marital status; 13 for having children. A statistically significant relation between depersonalization and gender (*r* = 0.078), marital status (*r* = 0.047), and children (*r* = 0.053) was found. A significant relation was also found between emotional exhaustion and children (*r* = 0.048). The results showed that being male, being single or divorced, and not having children were related to the highest levels of burnout in nurses. Moreover, these relations could be accentuated by the influence of moderator variables (age, seniority, job satisfaction, etc.), which, in combination with the previously mentioned significant relations, should be evaluated in the design burnout risk profiles for nursing professionals.

## 1. Introduction

The term ‘burnout’ was coined in the 1970s to describe the physical and emotional exhaustion that workers may experience on the job, especially those who provide some type of service to others [1,2]. Burnout is a process in which workers are continuously subjected to stressors that they find themselves unable to cope with. This makes them feel exhausted, lacking in energy, and mentally fatigued [3].

Maslach & Jackson described burnout as having three dimensions, which can be evaluated by the Maslach Burnout Inventory (MBI) [4]. These dimensions are: (i) emotional exhaustion (EE), related with the sensation of physical overexertion and mental weariness; (ii) depersonalization (D) or the presence of negative and cynical attitudes towards patients and colleagues; and (iii) low personal accomplishment (PA), reflecting the tendency to assess oneself negatively in relation to job performance and perceived general competence [4,5,6]. 

Although other instruments have been devised for burnout assessment [7,8] the questionnaire most frequently used and accepted by researchers is the Maslach Burnout Inventory (MBI) [9,10]. In fact, there are numerous psychometric studies that support this type of evaluation and modelization of the syndrome [11,12].

Although burnout is present in a wide range of occupations, health professionals (particularly nurses) are one of the occupational groups that is most at risk of developing it, due to the characteristic of their work and expending most of their working time in contact with the patients [13,14,15]. As showed in some studies, the prevalence of emotional exhaustion is around 30% in oncology and emergency nurses, while depersonalization prevalence is 15% in oncology nursing or 36% in emergency nursing [16,17].

In addition to affecting the mental and physical health of workers, burnout has repercussions on the quality of care and services provided. It also favors workplace absenteeism and even premature departures from the nursing profession [18,19,20].

For the effective prevention of this syndrome, it is crucial to identify the occupational, sociodemographic, and psychological variables related to its development [15,21,22]. To date, the focus has been on occupational variables [23], which are generally regarded as being most related to the syndrome—such as work experience, monthly income, working hours or job security [24,25]. However, there are few research studies that are exclusively centered on sociodemographic factors and their results are contradictory.

For example, certain research studies found no relation between burnout and the gender of nursing professionals [26,27], whereas other authors claim precisely the opposite [28,29]. Similarly, there are also contradictory findings for the correlation between burnout and marital status. Various studies claim that being single or married is unrelated to the three burnout dimensions [30,31]. In contrast, according to other research, workers who are single present higher levels of burnout [32,33], whereas other studies claim that being married is correlated with the syndrome [34,35]. There is also controversy in regard to having or not having children. Whereas certain authors say that this variable has no relation on burnout development [36,37], others have found a significant relation between the two. Some studies claim that nurses without children have higher levels of burnout [6,38] that nurses with children have higher levels of burnout [39].

As shown above, it is not clear the relation that these sociodemographic variables (gender, marital status, and having children) may have in nursing burnout, because some studies inform about a positive correlation while others inform of a negative correlation or other authors said that there is no relation. Similar contradictions regarding the relation between burnout and other variables in nursing professionals have been addressed and clarified with meta-analytic studies as, for example, occupational variables (job seniority, professional experience, job satisfaction, specialization or work shift) or sociodemographic variables (age) [23,40]. To our knowledge, no meta-analyses has been done to clarify the shown controversy relation between nurses’ burnout and gender, marital status, and having children, which has been clarified with meta-analytic studies in teachers and police officers [41,42]. Thus, the study of the potential relation of these variables with nurses’ burnout using a meta-analysis can provide valuable insights into whether they are relevant and should be included in nurses’ burnout risk profiles. Research of this nature would also provide the groundwork for the design and implementation of prevention programs or interventions for nurses who match the risk profile, to avoid the effects that burnout has for nurses, patients, and hospital institutions.

The objectives of this study were the following: (a) to perform a meta-analysis of the effect size of the correlation between sociodemographic factors (i.e., gender, marital state, and children) and the three burnout dimensions, measured with the MBI in nursing professionals; (b) to examine the moderator variables that could explain the heterogeneous results obtained by previous research. Thus, the question that guided the meta-analysis was: in nurses, what are the effect sizes (correlation) between the burnout dimensions and the sociodemographic factors gender, marital status, and having children?

## 2. Materials and Methods

### 2.1. Data Sources and Inclusion Criteria

A meta-analysis was performed, according to Preferred Reporting Items for Systematic Reviews and Meta-Analyses (PRISMA) statement, which refers to provide an investigation question, specifying studies selection criteria, informing about search strategy and information sources and detailing the procedure related to review validity and replicability. Different strategies were used to identify the higher number of primary studies [43].

First, the following electronic databases were consulted: CINAHL, CUIDEN, Dialnet, Psicodoc, ProQuest Platform (ebrary e-books, Health and ProQuest Deep Indexing (Medical), PsycARTICLES, PsycINFO, ProQuest Health & Medical Complete, ProQuest Deep Indexing, Medline), OVID SP Platform (Global Health, Ovid Nursing Database, ERIC), and Scopus. The search equation was (“Maslach Burnout Inventory” OR “MBI”) AND “nurs*”, without using any search restriction. Secondly, the references of other systematic reviews and meta-analysis about nurses’ burnout were consulted. Thirdly, gray literature was obtained in Google Scholar, ProQuest Dissertations & Theses, and TESEO databases. The Science Citation Index was then accessed to find studies citing the work thus identified. Finally, the references from the included studies, were also checked. The search was performed in May 2018 without any time restrictions.

The studies inclusion criteria were the following: (a) empirical quantitative; (b) use the MBI to assess burnout syndrome; (c) nursing professionals sample; (d) include correlation between sociodemographic variables (gender, marital status, and children) and one of the MBI dimensions, or the inclusion of statistical information for effect size calculation; papers in Spanish, English, French, Italian, or Portuguese were included. The MBI was used as an inclusion criteria because is the most widely accepted and used instrument for burnout measurement [2]. Other instruments were not included because the burnout dimensions are different and correlation comparison would not be adequate. The references of the meta-analysis are available upon request from the corresponding author.

To ensure the reliability of the process the search process was conducted by two members independently. In case of discrepancies, a third member of the team, that was blind to the other members’ decision, was consulted. From the papers finally selected, backward and forward citation checking was performed.

### 2.2. Critical Reading

The methodological quality was evaluated by the checklist suggested by Ciapponi [44], using the items corresponding to the studies internal validity: numbers 2, 3, 4, 5, 6, 11, 12, 13, 14, 15, 16, 17, and 18.

The initial search produced a total of 27,357 studies, which were reduced to 20,676 once duplicate titles were eliminated. However, after the titles of the articles were read, this number decreased to 5291. The abstracts of this research were then read, which led to 256 studies after excluding studies that did not use the MBI, did not have a nursing sample or were not quantitative and empirical. After reading the full text and excluding the articles that did not provide statistical data for the meta-analysis, the final number of studies was 78, more specifically, 57 for gender, 32 for marital status, and 13 for children.

**Figure 1:**
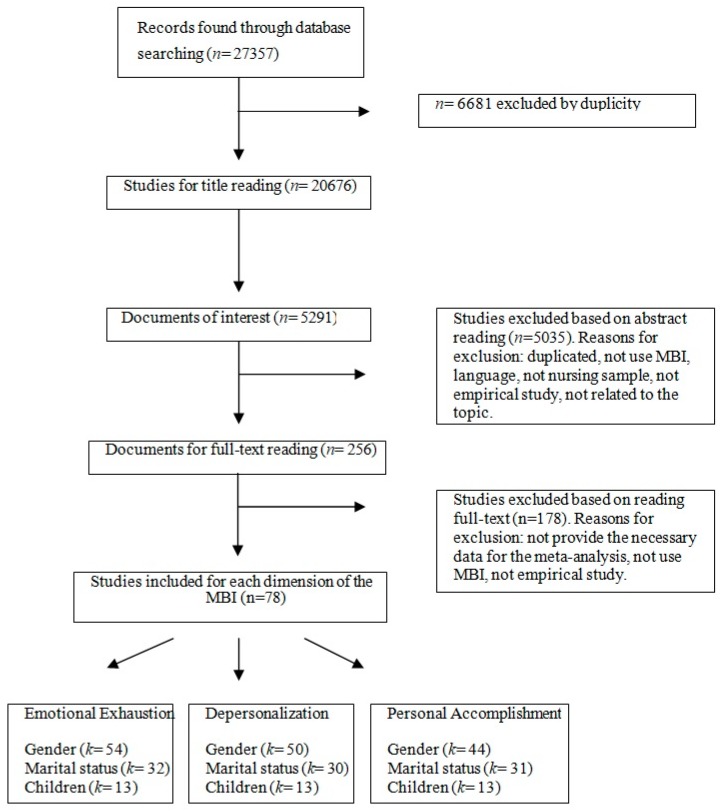
Flow chart for selection of included studies in meta-analysis. *k*: sample of studies; MBI: Maslach Burnout Inventory; *n*: number of studies.

These papers provided data for 54 samples of the correlation between gender and emotional exhaustion; 50 samples of gender and depersonalization; and 44 samples of gender and low personal accomplishment. In regard to marital state, there were 32 samples for its relation to emotional exhaustion; 30 for its relation to depersonalization; and 31 for its relation to low personal accomplishment. In reference to children, there were 13 samples for each of the burnout dimensions (see Figure 1). The total number of subjects in the meta-analysis was 35,925 for gender, 9957 for marital status, and 6125 for children.

### 2.3. Coding of Variables

A coding manual was written as part of the study, which is available upon request from the corresponding author. It records variables that could potentially moderate the correlation effect size [45]. The variables included in the coding manual were classified as substantive, methodological, or extrinsic [46]. Substantive variables were: age (mean and standard deviation); gender (women percentage); marital status (percentage of subjects living with a partner); children (percentage of subjects with children); professional experience (mean and standard deviation of the length of time working as a nurse); job seniority (mean and standard deviation of the length of time working at the current job); job satisfaction (mean and standard deviation of job satisfaction; MBI scores (mean values and standard deviation of each dimension).

Methodological variables were: sample size; Cronbach’s Alpha Coefficient each dimension of the MBI; MBI (original or adaptation); MBI language (Spanish/English/other); research design (experimental/quasi-experimental/observational); number of centers where data were collected (one or more than one); sampling technique (random/convenience); and response rate.

The extrinsic variables were: type (JCR journal/non-JCR journal/PhD dissertation); continent where the research was performed (Africa/America/Asia/Europe); and publication date.

The reliability of the codification process was evaluated in a random sample of studies (20%) by two researchers who were not directly involved in the study. Codification reliability was found to be satisfactory. The mean degree of convergence in continuous variables was obtained with the intraclass correlation coefficient, obtaining a value of 0.94 (minimum = 0.85; maximum = 1) while Cohen’s Kappa coefficient was used for the mean degree of convergence in categorical variables, 0.93 (minimum = 0.83; maximum = 1).

### 2.4. Effect Sizes

Effect size was determined as the Pearson bivariate correlation between each of burnout dimension (emotional exhaustion, depersonalization, and personal accomplishment) measured with the MBI and the next sociodemographic factors: gender (0 = female; 1 = male); marital status (0 = married/living with a partner; 1 = single/divorced); children (0 = with children; 1 = without children), following the guidelines in Rosenthal [47]. Thus, nine independent meta-analyses were done between the three burnout dimensions and the three socio-demographic variables.

### 2.5. Statistical Analysis

Independent meta-analyses were carried out to examine the correlation between sociodemographic factors and MBI dimensions. Previous to the calculation of the mean effect sizes (Pearson’s correlation), an exploratory analysis was performed to determine normality and detect the existence of outliers. To establish variances and fit the distributions to the normality curve, Pearson’s correlation was converted to Fisher’s *Z* before making the meta-analytic calculations. The next step was to convert Fisher’s *Z* to Pearson’s *r* in order to show the mean effect sizes with their associated confidence interval (CI) between the sociodemographic factors (gender, marital status, and children) and each of the MBI dimensions [45].

The *Q* text was used to assess the presence of heterogeneity and the *I*^2^ index was used to evaluate the heterogeneity degree of the mean effect sizes [45]. In regard to the relations of gender, marital status, and children to the MBI dimensions, random-effect models were applied [45,48].

Egger test was used to assess the publication bias, and a sensitivity analysis to judge the impact of each study on the mean correlations obtained [45]. To analyze the impact of the continuous moderator variables on the mean effect size, simple meta-regressions were perfomed. To compare the influence of the different subgroups of each categorical variable (MBI language, MBI type, sampling etc.) in the effect size, ANOVAs were used [49]. Statistical analyses were done with the software Comprehensive Meta-Analysis 3.0 (Biostat, Englewood, NJ, USA), and the statistical software package SPSS, version 22 (IBM, Armonk, NY, USA).

## 3. Results

### 3.1. Characteristics of Studies in Sample

The sample was *n* = 78 studies. Regarding the precedence of the studies, the 44% were done in Europe, the 34% in America and the 22% in Asia. 93% of the studies were observational and 88% were journal papers while the 12% where doctoral theses. The 27% of the studies were published in 2010, 2012, and 2013. Regarding the sampling method, 91% of the studies used convenience sampling.

### 3.2. Description of Effect Sizes (Pearson Correlations)

The mean correlations between gender and the MBI dimensions were the following: emotional exhaustion *r* = −0.014 (95% CI: −0.032, 0.003; *p* = 0.114; *k* = 54); depersonalization *r* = 0.078 (95% CI: 0.040, 0.115; *p* = 0.042; *k* = 50); and low personal accomplishment *r* = −0.004 (95% CI: −0.027, 0.018; *p* = 0.711; *k* = 44). The mean correlations between marital status and the MBI dimensions were the following: emotional exhaustion *r* = 0.014 (95% CI: −0.026, 0.054; *p* = 0.480; *k* = 32); depersonalization *r* = 0.047, (95% CI: 0.006, 0.088; *p* = 0.039; *k* = 30); and low personal accomplishment *r* = −0.006 (95% CI: −0.045, 0.033; *p* = 0.762; *k* = 31). Finally, the mean correlations between having children and the MBI dimensions were the following: emotional exhaustion *r* = 0.048 (95% CI: 0.016, 0.081; *p* = 0.003; *k* = 13), depersonalization *r* = 0.053 (95% CI: 0.003, 0.103; *p* = 0.036; *k* = 13); and low personal accomplishment *r* = 0.012 (95% CI: −0.048, 0.071; *p* = 0.703; *k* = 13). The effect sizes found between all the variables and the burnout dimensions were small.

The homogeneity analysis for gender, marital status, and having children found variability in the three mean effect sizes (correlations). The *Q* test was thus significant for the three MBI dimensions in relation with gender, marital status, and having children. The *I*^2^ index showed the lower level of heterogeneity for having children and emotional exhaustion (31.37%) and the highest for gender and depersonalization (84.48%). The *Q* test and *I*^2^ values indicate the need to analyze the variables that may be moderating the heterogeneity reflected in some of the previously mentioned correlations.

Egger’s analysis did not show publication bias for any burnout dimension in relation to gender: emotional exhaustion (*p* = 0.075), depersonalization (*p* = 0.085), and low personal accomplishment (*p* = 0.80). The same occurred in the case of marital status, emotional exhaustion (*p* = 0.24), depersonalization (*p* = 0.25), and low personal accomplishment (*p* = 0.29), as well as in the case of the variable, having children: emotional exhaustion (*p* = 0.10), depersonalization (*p* = 0.22), and low personal accomplishment (*p* = 0.33).

### 3.3. Analysis of Moderator Variables

An analysis of moderator variables was done to, as previously indicated, examine the moderator variables that could explain the heterogeneous results obtained by previous research. The values of β and *r*^2^ values that are shown in the tables refer, respectively, to the results of the regression analysis and to the proportion of the variance explained by the moderator variable when the other variables are constant. Only the moderator variables that were statistically significant are shown in the tables.

The analysis of the correlation between gender and emotional exhaustion showed that the following substantive moderator variables were statistically significant: standard deviation of the age of the sample (*p* < 0.05); mean and standard deviation of depersonalization (*p* < 0.01); mean and standard deviation of low personal accomplishment media (*p* < 0.01), and standard deviation of professional experience (*p* < 0.01). The following methodological and extrinsic moderator variables were also statistically significant: Cronbach’s Alpha Coefficient of low personal accomplishment (*p* < 0.01) and the year of publication (*p* < 0.01) (Table 1 and Table 2).

For the correlation between gender and depersonalization, the following substantive moderator variables were found to be statistically significant: mean and standard deviation of job seniority (*p* < 0.01). Regarding the correlation between gender and low personal accomplishment, the analysis showed that the following substantive moderator variables were statistically significant: sample percentage with children (*p* < 0.05) and standard deviation of job satisfaction (*p* < 0.05) (Table 1 and Table 2). The variance of the effect sizes between the burnout dimensions and nurses’ gender, was most affected by job seniority and having children.

Substantive moderator variables regarded as statistically significant for the relation between marital status and emotional exhaustion included mean value and standard deviation of low personal accomplishment (*p* < 0.01). The correlation between marital status and depersonalization was moderated by the sample percentage with children (*p* < 0.05) and the methodological moderator, response rate (*p* < 0.01). Finally, substantive moderator variables that were shown to be statistically significant for marital status and low personal accomplishment were percentage of the sample with children (*p* < 0.01) and the mean value of job seniority (*p* < 0.05) (Table 1 and Table 2). Having children and personal accomplishment mean score were the variables that most affected the variance of the effect sizes between the burnout dimensions and nurses’ marital status.

The analysis of the correlation between percentage of the sample with children and depersonalization showed the following methodological moderator variables to be statistically significant: Cronbach’s Alpha Coefficient of depersonalization (*p* < 0.05), language of the MBI (*p* < 0.01), and type of sample (*p* < 0.05). In regard to the correlation between percentage of the sample with children and low personal accomplishment, the substantive moderator variable of gender was found to be significant (*p* < 0.05) as well as the methodological moderator variable, language of the MBI (*p* < 0.05) (Table 1 and Table 2). The variance of the effect sizes between the burnout dimensions and having children was most affected by gender and low personal accomplishment Cronbach’s alpha coefficient.

Finally, multiple regression models were established to obtain explanatory models of effect size (correlations) variation [50]. These multiple regressions are not included in this paper because they did not meet the inclusion criteria (i.e., theoretically relevant and statistically significant moderator variables), given the fact that there were not a sufficient number of studies for a good fit to the model.

## 4. Discussion

The aims of the study were to calculate the effect size of the correlation between gender, marital state, and having children and the three burnout dimensions and to examine the moderator variables that could explain the heterogeneous results. The effect sizes found were low [45]. Compared with similar studies, higher correlations were found between occupational factors like job satisfaction and specialization with the burnout dimensions [23], and between age and depersonalization [41].

### 4.1. Correlations between Sociodemographic Variables and the MBI Dimensions

The results of our analysis showed a positive and statistically significant correlation between gender and depersonalization. More specifically, male nurses of the included studies seemed to have a greater tendency to show negative attitudes towards patients and their colleagues at the workplace. This evidently affects interpersonal relations within the medical care team as well as interprofessional relations between different teams. It also has a negative impact on the quality of service provided by the healthcare centers where these nurses work [29,36].

The correlation between marital status and depersonalization was positive and statistically significant. Subjects without a partner had higher levels of depersonalization. This could be due to the fact that the family environment of a couple life style is a factor that provides security and support, and which protects the subject from developing impersonal, cynical, and negative attitudes towards colleagues at the workplace. These results coincide with those reported by other authors [11,51,52].

Having children had a low positive though significant correlation with levels of emotional exhaustion and depersonalization in nursing professionals. Being childless appears to be related to higher levels of emotional exhaustion and depersonalization, which could mean that having children protects nurses from increased levels of these burnout dimensions. It seems that the responsibility of raising children does not accentuate, but rather reduces, the emotional overload and sensations of overwork that nurses often experience. In addition to gender, marital status, and children, similar studies have identified the correlation between burnout and other variables such as job satisfaction, specialization and age [23,41].

The value of the previously mentioned relations may be reinforced, depending on the values of moderator variables. It is necessary to take into account that these variables do not only influence nurses’ burnout individually, and can be moderators of the relationship between other variables and burnout. That is, the union of some of them can constitute a profile associated with the increase or decrease of the incidence of the syndrome. As shown in the results, being male, being single, and not having children is correlated with higher levels of burnout. However, the relations between these variables and the burnout is moderated by other variables and can be stronger when men have less than 10 years of working experience, or the single person do not have children or when the person without children is a man.

### 4.2. Analysis of Moderator Variables

Accordingly, the correlation between gender and emotional exhaustion appears to be moderated by age and professional experience as well as by the depersonalization and low personal accomplishment levels in nurses. This correlation was stronger when the dispersion in the variables of age and professional experience was eliminated. In this context, the subjects were between 30–40 years of age and had been working as nurses for less than 10 years [53,54].

The correlation between gender and emotional exhaustion was also higher in the case of nurses with high levels of the other burnout dimensions [55,56]. It may also be moderated by certain methodological and extrinsic variables, psychometric indicators of the tests used, and the year of publication. When the reliability of the tests was greater and the publication date was more recent, our results confirmed that that gender does not necessarily have to be regarded as a risk factor of emotional exhaustion. The absence of relation between emotional exhaustion and gender agree with other studies [22,23].

The variables that moderated the correlation between gender and depersonalization were the mean value and variability of job seniority. The relation between gender and depersonalization was quite strong, and should be considered in the case of men who have been working less than 10 years in their current post [57]. It should also be highlighted that in the case of nurses with children, there was a strong correlation between gender and low personal accomplishment with female nurses reporting higher levels of fulfillment [13].

The correlation between marital status and emotional exhaustion was stronger for those nurses with high low personal accomplishment scores. Unmarried nurses (single/divorced) could potentially show higher emotional exhaustion levels [33]. The variables, having children and response rate, negatively moderated the correlation between marital status and depersonalization, which was stronger in the cases of nurses with children. In nurses with children, who are married or living with a partner, there was a higher risk of depersonalization at work [58]. Furthermore, since the correlation between marital status and low personal accomplishment had very low values, it would not be relevant in a possible risk profile for burnout. Nevertheless, its relevance could increase in the case of nursing professionals without children and with greater job seniority since, of the two groups considered, single or divorced subjects felt less fulfilled [59,60].

The variables that moderated the correlation between children and depersonalization were methodological. This correlation was stronger when the reliability of the tests was greater and when adaptations of the MBI were used. This has also been observed by other authors [6,21,51]. Based on the results of our study, the correlation between having children and feeling more or less fulfilled was not significant. However, this correlation could become stronger when the subjects were female and when the test was administered in Spanish [39].

### 4.3. Recommendations and Suggestions for Hospital Managers and Nursing Professionals

As shown in the results, variables such as gender, marital status, and having children are related to nursing burnout. Regarding the results implications, nurses’ managers should take into account that male nurses that are single or divorced and who do not have children may be more prone to burnout. Thus these nurses should be a target population for burnout treatment and prevention programs and for hospital initiatives to promote better work wellbeing. In addition, they should assess the need to carry out reception programs for new nurses with these characteristics. Nursing professionals should be aware of the physical, mental, and emotional effort required by their profession and request, if necessary, support among their peers and other professionals to explain how they feel or to request interventions for burnout treatment or prevention.

Future research should assess the effectiveness of interventions for burnout prevention and treatment in nurses and analyze other variables that may be related with burnout syndrome.

### 4.4. Study Limitations

This study had limitations. Firstly, the number of studies included for some meta-analysis (having children) were low because the researchers have been focusing their attention in other variables. Secondly, those variables with a low number of studies whose results pertain to the moderation of burnout correlations should be taken into account, but with certain precaution. In fact, they should continue to be studied so that, in the future, more meta-analytic studies can be performed. Finally, the publication date was not restricted to be able to find the higher number of studies as possible.

## 5. Conclusions

In conclusion, there was a significant correlation between gender and depersonalization, being the values higher in men. Marital status also had a significant association with depersonalization with higher values in single or divorced subjects. Regarding children, a significant correlation between emotional exhaustion and depersonalization were found. Nurses without children had higher scores for both dimensions. In other words, being male, being single or divorced, and being childless seems to be related to higher levels of burnout in nursing professionals.

In addition, the correlations analyzed can be accentuated by different moderator variables. It would thus be necessary to consider certain special contexts. For example, emotional exhaustion seemed to increase in nurses 30–40 years of age, who had less than 10 years of professional experience, and there were higher levels of depersonalization in men with less than 10 years of experience in their job. Particularly, nurses who are married or living with a partner and without children were found to have a greater risk of depersonalization. 

These variables should be taken into account in the design of risk profiles for burnout in nursing professionals. This would help to implement prevention programs, such as nurses’ support groups or mindfulness for nurses who at most at risk of developing burnout, and in this way, some of its more serious consequences could be avoided.

## Figures and Tables

**Table 1:** Weighted simple regression of quantitative moderator variables.

Variables	*k*	β	*Q*_R_	*Q*_E_	*R* ^2^
Gender					
*Emotional Exhaustion*					
SD mean age	34	−0.0140	5.39 *	33.61	0.138
Mean depersonalization	35	0.0069	10.08 **	30.59	0.247
SD depersonalization	35	0.0095	10.03 **	30.64	0.246
Mean personal accomplishment	34	0.0016	9.25 **	30.17	0.234
SD personal accomplishment	34	0.0062	7.39 **	32.00	0.177
SD job seniority	15	−0.0480	9.20 **	12.86	0.417
Alpha coefficient PA	21	−0.6226	4.37 **	22.13	0.164
Publication year	54	−0.0050	7.26 **	53.72	0.119
*Depersonalization*					
Mean job seniority	5	−0.0368	8.19 **	15.87 **	0.340
SD job seniority	5	−0.0481	9.65 **	14.41 **	0.401
*Personal Accomplishment*					
Children	9	−0.0030	4.79 *	10.87	0.305
SD job satisfaction	6	0.1847	4.01 *	12.49 *	0.243
Marital state					
*Emotional Exhaustion*					
Mean personal accomplishment	21	0.0056	8.04 **	24.98	0.243
SD personal accomplishment	21	0.0222	7.75 **	22.68	0.254
*Depersonalization*					
Children	5	−0.0057	4.02 *	11.14 **	0.265
Response rate	24	−0.0035	9.29 **	26.07	0.262
*Personal Accomplishment*					
Children	8	0.0042	7.40 **	20.02 **	0.269
Job seniority	5	0.0202	4.00 *	2.34	0.630
Children					
*Depersonalization*					
D Cronbach’s alpha coefficient	5	0.8081	4.36 *	6.38	0.405
*Personal Accomplishment*					
Gender	7	0.0047	4.48 *	5.89	0.432

Note: *k*: number of studies; β: standardized regression coefficient; *Q*_R_: value for the inter-group effects; *Q*_E_: statistical value of the homogeneity of the effect size within each group; *R*^2^: proportion of the variance explained by the moderator variable; DT: standard deviation. * *p* < 0.05, ** *p* < 0.01.

**Table 2:** Weighted ANOVAs for the moderator variables of the effect size of burnout.

Variables	*k*	*r*	95% CIs	ANOVAs	*r* ^2^
Children					
*Depersonalization*					
MBI language					
Spanish	5	0.026	(−0.021, 0.073)	Q_B_(2) = 9.398 ** Q_w_(10) = 11.140	0.457
English	3	−0.026	(−0.232, 0.183)		
Other	5	0.126	(0.078, 0.173)		
Sample					
Randomized	3	−0.031	(−0.103, 0.042)	Q_B_(1) = 6.485 *	0.266
Convenient	10	0.083	[0.034, 0.132]	Q_w_(12) = 13.119	
*Personal Accomplishment*					
MBI language					
Spanish	5	0.061	(−0.007, 0.129)	Q_B_(2) = 8.454 * Q_w_(11) = 20.449 *	0.292
English	3	−0.128	(−0.233, −0.020)		
Other	5	0.015	(−0.106, 0.136)		

Note: CI: confidence interval; *k*: number of studies; *r*: mean effect size; *Q*_B_: inter-category *Q*value; *Q*_w_: Intracategory *Q*value; *r*^2^: proportion of the variance explained by the moderator variable. * *p* < 0.05, ** *p* < 0.01.
